# Strain evolution and confinement effect in InAs/AlAs short-period superlattices studied by Raman spectroscopy

**DOI:** 10.1038/s41598-022-26368-8

**Published:** 2023-01-04

**Authors:** Yinan Zhao, Kechao Lu, Jinshan Yao, Jiqiang Ning, Baile Chen, Hong Lu, Changcheng Zheng

**Affiliations:** 1 Division of Natural and Applied Sciences, Duke Kunshan University, Kunshan, 215316 People’s Republic of China; 2 National Laboratory of Solid State Microstructures, Department of Materials Science and Engineering, College of Engineering and Applied Sciences, Nanjing University, Nanjing, 210093 People’s Republic of China; 3 Department of Optical Science and Engineering, School of Information Science and Technology, Fudan University, Shanghai, 200438 People’s Republic of China; 4 School of Information Science and Technology, ShanghaiTech University, Shanghai, 201210 People’s Republic of China; 5 Jiangsu Key Laboratory of Artificial Functional Materials, Nanjing University, Nanjing, 210093 People’s Republic of China; 6 Shanghai Engineering Research Center of Energy Efficient and Custom AI IC, Shanghai, 201210 People’s Republic of China

**Keywords:** Nanoscale materials, Surfaces, interfaces and thin films

## Abstract

Raman spectra of two series of InAs/AlAs short-period superlattices were measured at room temperature to investigate the impact of strain on the phonon modes taking into consideration the confinement effect and interface mode. The evolution of strain in the InAs layer and the AlAs layer was studied in (InAs)_2_/(AlAs)_2_ superlattices grown at various temperatures (400–550 °C). While the strain existed in the AlAs layer remained almost constant, the strain in the InAs layer varied significantly as the growth temperature increased from 500 to 550 °C. The confinement effect on the optical phonons was analyzed based on results from (InAs)_n_/(AlAs)_n_ grown at 450 °C (n = 2, 3, 4, and 5). Additionally, the confinement effect was found to be stronger in shorter periods with higher interface quality. The interface phonon modes were resolved between the longitudinal optical and transverse optical phonon modes, which assist in the rough estimation of the thickness of the layers. The disorder-activated acoustic phonon modes at the low-frequency side were also addressed.

## Materials and methods

The InAs/AlAs short-period superlattice (SPS) grown by molecular beam epitaxial (MBE) has attracted continuous interest since its ordered structure leads to unique optical and electronic properties^1,2^, such as high electron mobility^3^ and large tunable bandgaps^4^. Therefore, InAs/AlAs superlattice has a promising future in the field of device applications, including channel composition modulated transistor (CCMT)^5^ and infrared photodetectors^6^. It has been reported that superlattices can have the possibility of tailoring the electronic and optical properties when components are under considerable strain^7^. It is important to investigate InAs/AlAs superlattice in terms of its nanostructure and the quantum dots formed^8^.

Over the past years, many achievements and advancements have been made in the study of InAs/AlAs superlattices and materials with similar structures, especially in the optical characterization field. For example, the region around 360 cm^−1^ in Raman spectra is due to the vibration of AlAs, while peaks located in the 230–250 cm^−1^ region arise from the vibration of InAs^3^. The peak assignments were made based on the Raman modes of InAs and AlAs crystals, respectively. At room temperature, the InAs longitudinal optical (LO) phonon mode is located at 238.6 cm^−1^ and the transverse optical (TO) mode is at 217.3 cm^−1^ ^9^. In the AlAs region, the LO and TO phonon modes were determined at about 400 cm^−1^ and 363 cm^−1^, respectively^10,11^. The dependency of strain relaxation on the period was studied by controlling the number of monolayers of either InAs or AlAs^7^. More recently, the superlattices made of (InAs)_n_/(AlAs)_n_ exhibited bandgap-tunable property by adjusting the layer thickness, indicating that the cut-off wavelength of such material can be finely controlled^4^. As to other similar structures, the impact of different polarization configurations on the Raman peaks was studied with InAs/GaSb superlattices and several new modes were found^12^. Raman scattering was measured for (AlAs)_n_/(GaP)_n_ and (AlAs)_n_/(AlP)_n_ (n = 2, 3, 4, 5) to confirm the contribution of the confinement effect as well as the interface phonons on the peak position shift^1^.

However, the impact of the growth temperature and period length on the strain within the InAs/AlAs superlattice structure is seldom studied using Raman spectroscopy. The Raman peak shift can not only reveal the variance of the lattice mismatch strain and the individual layer thickness, but the broadening of the Raman peak can also present the confinement effect and lead to an insight into the density of defects. In this article, we use confocal micro-Raman spectroscopy, a non-invasive optical method, for the characterization of the strain status in InAs/AlAs superlattices. Generally, due to the lattice mismatches between InAs and AlAs, the InAs layer is under compressive strain while the AlAs layer undergoes tensile strain^7^. We focused on the evolvement of the peaks concerning the growth temperature and period length (i.e., 2/2, 3/3, etc.), which is closely related to the changes in strain and confinement effect. Previous literature has found that the confinement effect can lead to redshift^8^, while the influence of strain depends on whether it is tensile or compressive^7,13^. The interface (IF) mode is resolved between the LO and TO phonon modes, and it also tends to follow the shifts of LO and TO modes.

## Experimental details

To develop a comprehensive understanding of the evolvement of strain and confinement effect in the InAs/AlAs superlattices using different growth methods and conditions, two groups of samples were grown by MBE on semi-insulating InP (001) substrates. An In_0.52_Al_0.48_As buffer layer was grown at 490 °C on the InP substrate after the native oxides on the substrate were removed by thermally heating the substrate up to 530 °C. The first group is a series of (InAs)_n_/(AlAs)_n_ (n = 2, 3, 4, 5) superlattices with different periods grown at the same temperature, 450 °C. The period number is designed to be 200 for the (InAs)_2_/(AlAs)_2_ superlattice (2/2), 133 for 3/3, 100 for 4/4, and 80 for 5/5, respectively, so that the total thickness of the active layer is kept about the same, ~ 255 nm. The thicknesses of individual InAs and AlAs layers are estimated to be 6.48 Å and 6.05 Å for 2/2, 9.87 Å and 9.23 Å for 3/3, 13.32 Å and 12.44 Å for 4/4, 16.49 Å and 15.4 Å for 5/5, respectively, based on the XRD result^4^, which are used to estimate the thicknesses of the active layers, that is, for the 2/2 sample, (6.48 Å + 6.05 Å) × 200 = 250.6 Å. More details about the growth procedure can be found elsewhere^4^. The second group is a series of (InAs)_2_/(AlAs)_2_ grown under six different temperatures ranging from 400 to 550 °C. As the superlattice structure can be greatly affected by the periods and the interface quality is likely related to the growth temperature, the two groups were designed for the overall investigation of strain and confinement effect. The Raman scattering measurement was performed at room temperature on a confocal micro-Raman system (Horiba, LabRAM HR evolution) equipped with a 100X objective (NA: 0.90), excited by a 532 nm laser line in a back scattering configuration. The laser power is ~ 410 μW. The spectral resolution of the system is better than 1 cm^−1^.

## Results and discussion

**Figure 1:**
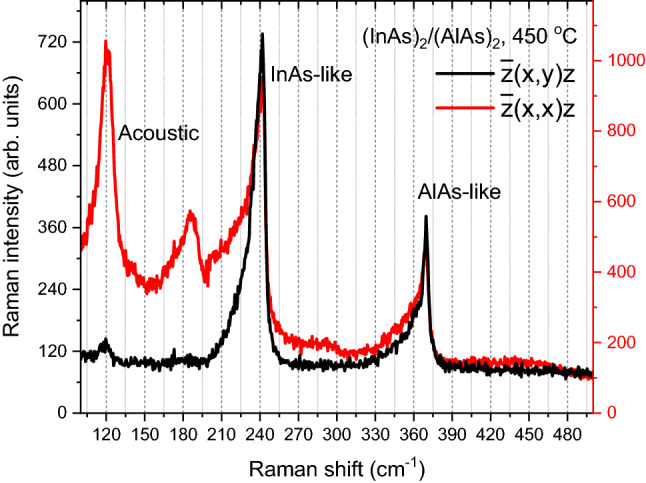
Raman spectra of (InAs)_2_/(AlAs)_2_ grown at 450 °C under different polarization configurations. The labels ‘acoustic’, ‘InAs-like’ and ‘AlAs-like’ indicates the respective phonon regions.

**Figure 2:**
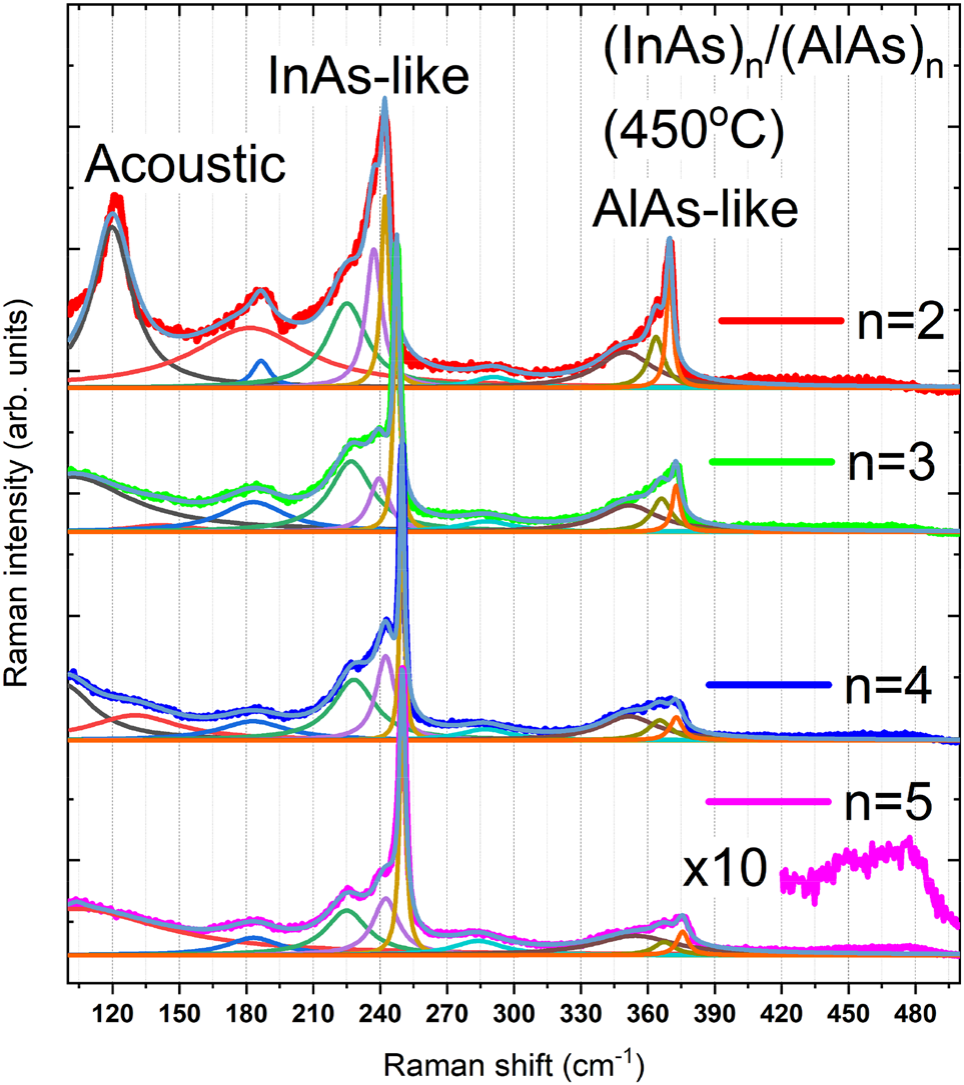
Raman spectra of InAlAs RA and (InAs)_n_/(AlAs)_n_ SPSs with n = 2, 3, 4, and 5 grown at 450 °C. The InAs-like, AlAs-like, and acoustic phonon regions are marked. Fitting results by Lorentz profile are shown. Three peaks are resolved in both InAs- and AlAs-like regions, respectively. The enlarged portion (X10) around 480 cm^−1^ for the n = 5 sample exhibits the second-order phonon signal.

Figure 1 shows the Raman spectra of (InAs)_2_/(AlAs)_2_ grown at 450 °C measured with different polarization configurations. In either InAs- or AlAs-like region, the most intensive peak is attributed to LO phonon while the shoulder on the low-energy side is assigned to the TO phonon^9,10^. Please note the InP-related Raman modes were not detected in all samples studied in this work. The Raman peaks present asymmetrical broadening between the LO and TO phonon modes in both InAs and AlAs regions. Such broadening is attributed to the IF phonon modes at the interface of the AlAs and InAs layers, respectively^8^. The fitting results are shown in Fig. 2 below. It is worth noting that we only observed the shoulders of Raman peaks in Fig. 1, and the folded optical phonon modes are not well resolved. As the folded phonons are usually shown in series on the low energy side, it has been reported that the even folded modes are detected in $\overline{z }(x,x)z$ configuration while the odd modes are shown in $\overline{z }(x,y)z$ configurations^14^, which is not the case here. Therefore, we assume that the folded optical phonons are very weak and cause little influence in our experiment, and the interface mode is required to fit the asymmetric Raman line shape. The peaks below 200 cm^−1^ correspond to the first zone folded longitudinal acoustic (LA) phonon^3^, because the folded LA peaks were observed in $\overline{z }(x,x)z$ configurations and vanished in $\overline{z }(x,y)z$ configurations^15^, according to a one-dimensional elastic continuum (Rytov) model^3,16^.

Figure 2 shows the original Raman spectra of the group of (InAs)_n_/(AlAs)_n_ superlattices with different periods. The Lorentz line shape was adopted to fit the overall asymmetric Raman peaks to obtain peak positions and full width at half-maximums (FWHMs), and hereinafter. The phonon confinement effect can be revealed by the broadening parameter in the integration of the Lorentz peaks^17^. Representative fitting results are depicted for n = 2 and n = 5 as examples. A weak broad peak around 285 cm^−1^ was introduced in the fitting, whose origin is unclear and further investigation is ongoing. The peaks for the second-order phonon modes are very weak so that only a second-order InAs-like LO phonon mode was found for n=5^9^, with a tenfold enlarged part inserted. The existence of the IF mode can lead to a broader peak in the second-order spectrum while the folded optical phonon modes such as LO_2_ and LO_4_ modes would result in a sharper peak. Hence, it is reasonable to assign the shoulder in the InAs- or AlAs-like region to the IF phonon mode^18,19^. The spectra show strong peaks at about 225 cm^−1^ and 247 cm^−1^ which can be attributed to the TO and LO modes of the InAs layers^9^. Peaks located at around 352 cm^−1^ and 373 cm^−1^ are due to the TO and LO phonons of the AlAs layers^20^. Interface phonons of the InAs and AlAs layers are found between the LO and TO phonon mode, responsible for the Raman peaks located at 237 cm^−1^ and 366 cm^−1^ respectively. It is worth mentioning that the theoretical calculation of the interface phonon-induced peaks are at 232 cm^−1^ and 350 cm^−1^ ^21^. The discrepancy of the peak positions for the different modes may be due to the strain and the confinement effect, which will be further addressed later. The folded longitudinal acoustic phonon peak nearly disappeared for other samples except for n = 2, which is similar to the findings by J. Bradshaw et al., that the peak observed around 115 cm^−1^ came from the bilayer sample and was absent for other samples^3^. Besides the two acoustic phonon modes (120 and 187 cm^−1^), we added an additional broad peak in the fitting process for obtaining better fitting results of the overall line shape, which may be caused by background signals in our measurements. The appearance of the acoustic Raman peak indicates the good periodicity of the n = 2 sample^1^ and the relatively high quality of the interface. The increase of FWHM of the IF mode with period may also imply an inverse relationship between spectral quality and the number of layers. Meanwhile, the increase of FWHM is closely related to the confinement effect.

**Figure 3:**
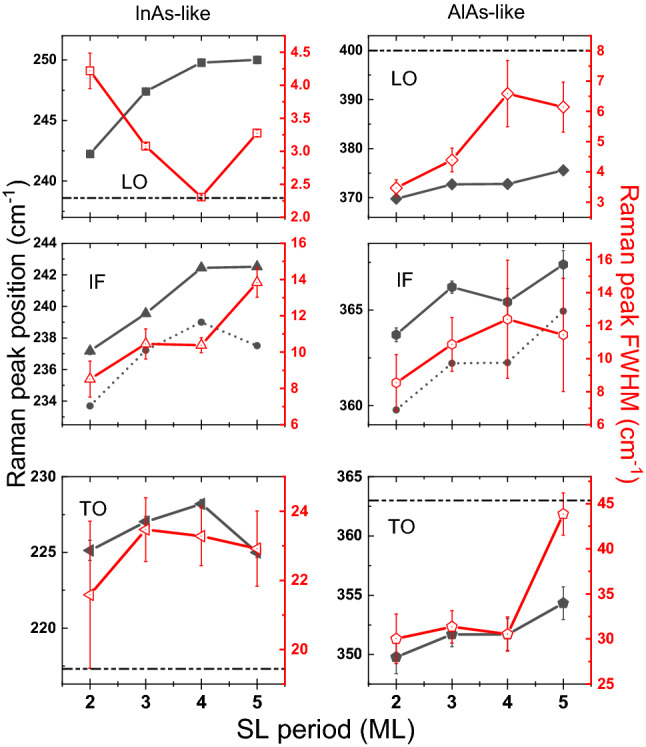
Peak positions and FWHMs of LO, IF, and TO phonon modes with error bars obtained from the fitting results concerning the periods. The horizontal dashed lines indicate the phonon frequencies for bulk InAs and bulk AlAs. The scatters connected by dashed lines in IF modes indicate the mid-position of LO and TO modes.

Since Raman spectra are highly sensitive to structural disorder and the Raman spectra measurements are performed under low laser excitations, defects may relax the momentum conservation rule which leads to the activation of non-center phonons^22^, the broadening of the Raman peak can be partly attributed to the confinement effect. Additionally, the polar optical phonon scattering plays a role in both the momentum and energy relaxation processes^23^. Since plane wave-like phonons cannot propagate through the lattice defects such as dislocations, vacancies or interstitials^17^ that existed in the layers, the weighting function introduced by the wave vector uncertainty shall result in the rapid decay of the phonon wave functions. The wave vector uncertainty is given by $\Delta q=\pi /L$, where *L* is the distance between dislocations^17^. As shown in Fig. 3, the peak broadening is observed for all the AlAs-like modes and InAs-like IF/TO mode, indicating that the confinement effect is nonnegligible for this series of samples. When the period *n* increases, all the peaks in InAs- and AlAs-like region have a tendency of blue shift, except the InAs-like TO mode. Therefore, we suppose the phonon modes are confined in both InAs and AlAs layers, and the blue shift of LO/IF/TO modes is attributed to the combined influence of confinement effect and strain. The sudden change of the peak position of the InAs-like TO mode may originate from the anomalous change in lattice constant for the n = 5 sample^4^. When the sample is grown with fewer periods, the quantum well would have a narrower “well width”, making the confinement effect stronger^24^. Since the confinement effect will lead to the redshift of phonon peaks^8,25^, the weakening of the confinement effect can cause blue shift instead, which corresponds to the increase in the number of monolayers in each period. However, comparing to the contribution of strain, the confinement effect has limited influence on the shift of peak positions. For InAs layers, the estimated LO phonon peak position shift resulted from the confinement is about 2 cm^−1^ ^26^, much smaller than the strain-induced shift. The confinement effect induced TO peak shift is almost negligible^26^. For AlAs layers, Tenne et al.^27^ suggested that the phonon peak position shift of AlAs can be mainly attributed to the strain due to the weak dispersion of the AlAs optical phonon.

**Figure 4:**
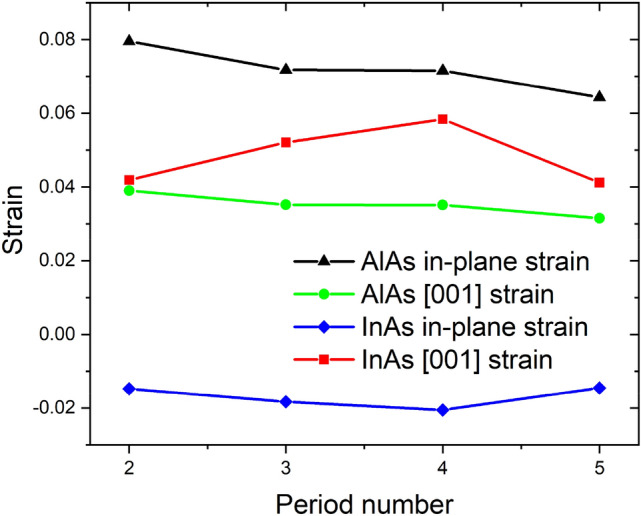
The calculated in-plane strain and the strain along the [001] growth direction for both InAs and AlAs layers in the (InAs)_n_/(AlAs)_n_ SPSs grown with different periods.

Due to the difference in lattice constant ($a_{AlAs} = 5.6611$ Å,^28^ $a_{InAs} = 6.0583$ Å ^29^) there exists biaxial compressive strain in InAs layers and tensile strain in AlAs layers, respectively, compared to bulk InAs and AlAs^7^. Without considering the confinement induced peak position shift, we can estimate the biaxial strain from the variance in the InAs-like and AlAs-like modes with the secular equation given by Cerdeira et al*.*^30^ and the strain can be obtained by the following equation:^31^

1 $$\omega_{TO} - \omega_{0} = \Delta \omega = \frac{p + q}{{2\omega_{0} }}\epsilon_{||} + \frac{q}{{2\omega_{0} }}\epsilon_{ \bot }$$

2 $$\epsilon_{ \bot } = \frac{{S_{11} }}{{S_{12} }}\epsilon_{||}$$

where $S_{11}$ ($1.945 \times 10^{ - 12} \,{\text{dyn}}^{ - 1} \;{\text{cm}}^{2}$ for InAs, $1.20 \times 10^{ - 12} \;{\text{dyn}}^{ - 1} \;{\text{cm}}^{2}$ for AlAs) and $S_{12}$ ($- 0.685 \times 10^{ - 12} \;{\text{dyn}}^{ - 1} \;{\text{cm}}^{2}$ for InAs, $0.39 \times 10^{ - 12} \;{\text{dyn}}^{ - 1} \;{\text{cm}}^{2}$ for AlAs) are elastic compliances for either InAs^30^ or AlAs^32^; $p$ and $q$ are phonon deformation potentials describing the change in effective spring constants induced by the strain; $\epsilon_{||}$ and $\epsilon_{ \bot }$ are the in-plane strain and strain along the [001] direction; $\omega_{0}$ and $\omega_{TO}$ are the strain-free Raman shift of optical phonon modes and strain-exist Raman shifts, respectively. The values of $p = - 2.053\omega_{0}^{2}$ and $q = - 2.623\omega_{0}^{2}$ for InAs are obtained from Ref.^27^. The phonon deformation potential of AlAs is approximated with the $p$ and $q$ values of GaAs^33^. The justification of such an approximation is offered in the work of Tenne et al.^27^ Similar method has been adopted by Tran et al., in estimating the strain-induced shift that existed in the InAs/InP superlattice without considering the confinement effect and the theoretical calculation agrees well with the experimental value^26^. In our case, the strain that existed in the superlattice structure is roughly estimated based on the Raman peak position shift and calculated from Eqs. (1) and (2). Figure 4 presented the variance of the strain with the increase in the period number.

Here the positive strain values indicate the presence of the tensile strain while the negative ones present the compressive strain. From Fig. 4, it is obvious that the strain that existed in the AlAs layers decrease with the increase of the period number. Taking the change of period into consideration, thicker layers may contribute to the partial relaxation of the strain^7^. This agrees with the strain variance estimated in AlAs layers. The determination of tensile and compressive is based on comparison with Raman peak positions of respective bulk materials, so it is possible for the InAs and AlAs layers to be compressed/stretched. Along the [001] direction, the period length (the total thickness of *n* InAs layers and *n* AlAs layers) of the superlattice obtained from the XRD results^4^ is larger than the direct addition of the lattice constant (*n *× 5.6611 + *n *× 6.0583), indicating that both InAs and AlAs layers in superlattice undergo tensile strain as calculated.

On the other hand, following the blue shift of LO and TO modes due to strain and confinement effect, the in-between IF phonon mode tends to shift with LO and TO at the same time. Ideally, if the thickness is the same for each layer, the peak position of the IF phonon should be midway between LO and TO modes, based on the electrostatic model^18^. However, in our experiments we find that the IF phonons are always closer to the LO mode as shown in Fig. 3. It might be explained by the Frohlich electron-phonon interaction^18^ and dielectric continuum model^27^. As coupling happens between the observed modes and the excitons, the difference in the thickness of InAs and AlAs layers can affect the IF mode peak position^18^. The dielectric continuum model has taken into account the confinement effect, and the frequency-dependent dielectric constant can be calculated based on^18^

3 $$\varepsilon_{1, 2} \left( \omega \right) = \varepsilon_{1, 2}^{\infty } \left( {\omega^{2} - \omega_{L}^{2} } \right)/\left( {\omega^{2} - \omega_{T}^{2} } \right)$$

where $\omega_{L}$ and $\omega_{T}$ are the frequency of LO and TO modes, respectively, and ω is the IF phonon frequency. The subscript 1 and 2 correspond to InAs and AlAs, respectively. Hence, taking the long-wavelength limit, it can be estimated from $\varepsilon_{1} d_{2} + \varepsilon_{2} d_{1} = 0$ or $\varepsilon_{1} d_{1} + \varepsilon_{2} d_{2} = 0$. If adopting the former relation, the ratio of layer thickness ($d_{1} /d_{2}$) is in the range of 1.05–2.13. The dependence of peak position on the period also shows that IF mode is not strictly localized at the interface^34^. Moreover, it has been reported that the asymmetric broadening feature of InAs- and AlAs-like regions is also related to the IF phonon mode^8,19^. The enlarging FWHM of the IF modes as *n* increases may indicate the reduced interface quality as well, consistent with the observations of acoustic phonon mode.

**Figure 5:**
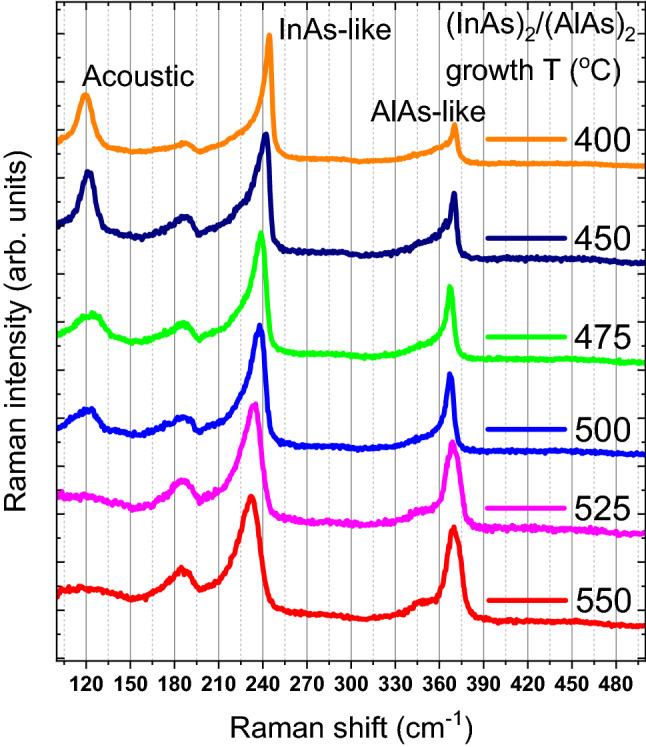
Raman spectra of (InAs)_2_/(AlAs)_2_ SPSs grown at different temperatures from 400 to 550 °C. The InAs-like, AlAs-like, and acoustic phonon regions are marked.

Figure 5 lists the Raman spectra of the other group of (InAs)_2_/(AlAs)_2_ samples grown at different temperatures from 400 to 550 °C. Similarly, the peaks around 230 cm^−1^ originate from the InAs-like modes and those around 370 cm^−1^ are from the AlAs-like modes. Each of the two regions is fitted by three Lorentz line shape peaks, attributed to TO, IF and LO phonons, respectively, from the left to the right. It can be seen that the InAs-like peaks show a redshift as growth temperature increases while the influence on the AlAs-like peaks is marginal. Meanwhile, the acoustic phonon mode around 120 cm^−1^ weakened with the increase in the temperature.

For growth temperature in the range of 400–475 °C, the FWHMs of the LO/TO/IF phonon peak of InAs and AlAs are small compared with the rest of the temperature points, indicating that the confinement effect is relatively weak and rare defects existed near the interface. In addition, the signal of the acoustic phonon induced peaks is quite strong in these Raman spectra, revealing the fine periodicity of the superlattice structure. A relatively low temperature is considered to be beneficial for growing InAs/AlAs SPSs with high interface quality. Higher temperatures can lead to a higher possibility for the occurrence of diffusion between layers. The broadening of the Raman peak is observed in both InAs-like and AlAs-like LO phonon modes with the increase in growth temperature while the width of the TO-induced Raman peak stays almost constant in the growth temperature range of 500–550 °C. Since the broadening of the TO phonon is not significant with the increase in the growth temperature, the effect of confinement is negligible on the TO phonon frequency shift^26^.

The density of the defects that exist in the superlattice structure may be deduced from the extent of the broadening of the Raman peak because phonon confinement effects are arising as a consequence of the loss of translational symmetry in the crystal caused by the high density of defects^35^ and causing the decrease of the distance between two adjacent defects and the enlargement of the wave vector uncertainty. It is worth noting that the width of the AlAs-like and InAs-like LO Raman peaks increases significantly when the growth temperature reaches around 525 °C, indicating that the density of defects varies dramatically at the 525 °C along the direction of the LO eigenvector. Peak broadening is also observed for the InAs-like IF phonon peak while the width of the AlAs-like IF phonon peak decreases for higher temperatures, indicating that the density of dislocations such as vacancies or interstitials increases at the InAs side of the interface but decreases at the AlAs side. The variance of the interface phonon mode peak width shows that the interface quality of both InAs and AlAs layers can be controlled by tuning the growth temperature, which resembles with the effect of growth temperature on the defect densities of Nitride materials^36^. Moreover, as the growth temperature increases, the Raman spectra of the SPSs become more similar to the spectrum of the random alloy^37^.

**Figure 6:**
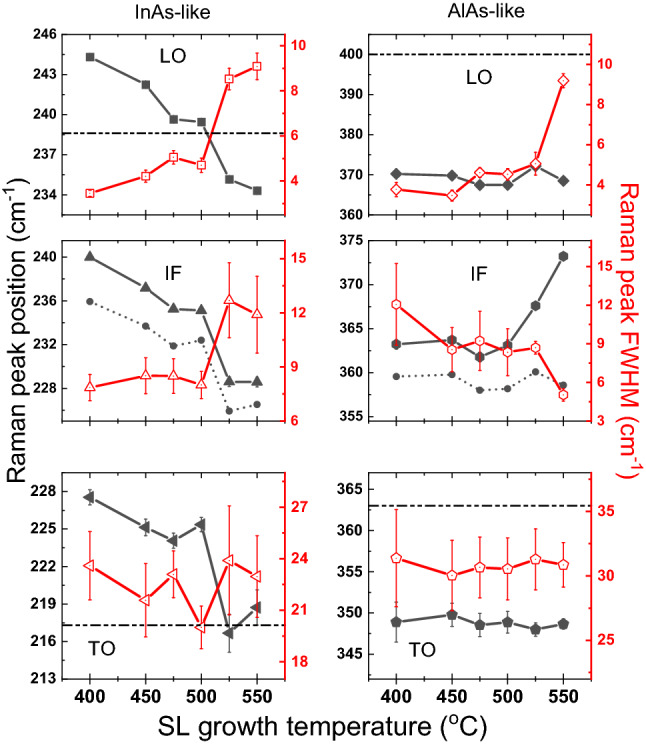
Peak positions and FWHMs of LO, IF, and TO phonon modes with error bars obtained from the fitting results concerning the growth temperature. The horizontal dashed lines indicate the phonon frequencies for bulk InAs and bulk AlAs. The scatters connected by dashed lines in IF modes indicate the mid-position of LO and TO modes.

**Figure 7:**
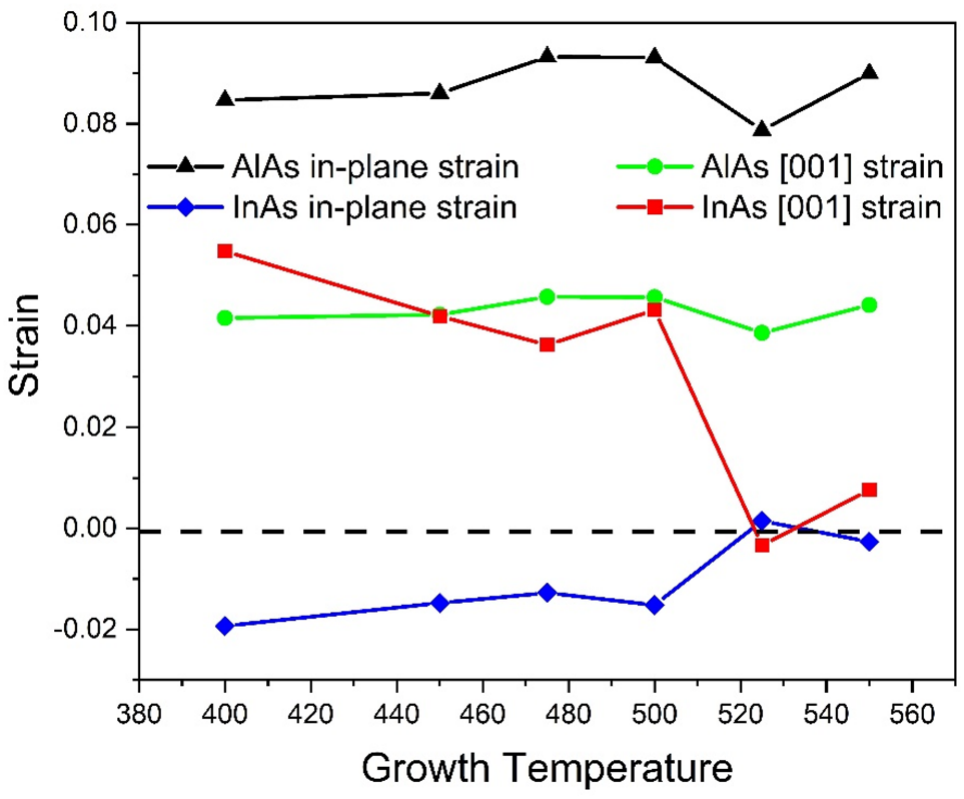
The calculated in-plane strain and the strain along the [001] growth direction for both InAs and AlAs layers in the (InAs)_2_/(AlAs)_2_ SPSs grown at different temperatures.

Extracted from the Lorentz fitting results, the Raman peak positions of InAs/AlAs-like LO/IF/TO phonon-induced Raman peaks vs. growth temperature are shown in Fig. 6. Significant redshift of peak position (about 10.4 cm^−1^) with the increase of superlattice growth temperature is observed for the three InAs-like phonon modes. Nakayama et al.^38^ have attributed the shift of the GaAs/In_x_Al_1−x_As superlattices LO mode Raman peak to the lattice mismatch-induced strains. Because both the strain and the confinement effect will result in the shift of the phonon frequency, the peak position shift of the TO phonon is used to estimate the strain since the influence from confinement is negligible as previously discussed. We also estimated the biaxial strain from the TO phonon peak positions based on Eqs. (1) and (2), and the result is shown in Fig. 7 below.

From Fig. 7, we can see that the strain of the AlAs layer almost stayed as a constant while the in-plane strain that existed in the InAs layer varied from compressive to almost relaxed (the strain along the [001] direction varied from tensile to almost relaxed) as the growth temperature increases. The calculated strain satisfies the coherent growth condition:^26^

4 $$a_{InAs}^{||} = a_{AlAs}^{||} = a_{InAs} \left( {1 + \epsilon_{InAs}^{||} } \right) = a_{AlAs} \left( {1 + \epsilon_{AlAs}^{||} } \right)$$

where $a_{InAs}$ and $a_{AlAs}$ are the lattice constant of bulk InAs and bulk AlAs mentioned above; $\epsilon_{InAs}^{||}$ and $\epsilon_{AlAs}^{||}$ are the in-plane strain of the superlattice estimated above; $a_{InAs}^{||}$ and $a_{AlAs}^{||}$ are the lattice constant of InAs/AlAs in strained layers. The coherent growth condition is mostly satisfied for different growth temperatures. For example, at the growth temperature of 525 ℃ the values of $a_{InAs}^{||}$ and $a_{AlAs}^{||}$ are 6.06 and 6.10 Å, respectively, indicating that the strain estimated previously is rather accurate. It is worth noting that the strain in both InAs and AlAs layers varied dramatically in the range of 500–550 °C, suggesting the wide strain tunability of the InAs-AlAs superlattice in such temperature range.

## Conclusion

According to the study of the Raman spectra of the two series of InAs/AlAs SPSs, one group of different periods and the other with various growth temperatures, the influence of strain and confinement effect are carefully analyzed. We calculated the specific strain for both groups. As the period number increases, a continuous decrease of the strain in the AlAs layers has been observed. For the growth temperature-dependent samples, it has been found that as the growth temperature increases, no obvious change occurred for the strain in the AlAs layer while the strain in the InAs layer varied radically in the range of 500–550 °C. The effect of confinement is found to be stronger in the sample set with different periods. Meanwhile, the confinement effect becomes weaker with the increase of periods. The interface modes are identified in the spectra in-between the LO and TO modes, which give the relative thickness of each layer and confirm the strain evolvement. The findings in this work may contribute to the fabrication of InAs/AlAs superlattices, providing additional evidence for the optimization of superlattice periods and growth temperatures in applications.

## Data Availability

The data and material generated during and/or analyzed during the current study are available from the corresponding author on reasonable request.

## References

[1] Nagano M, Sugie R (2005). Raman scattering in AlAs/GaP and AlAs/AIP strained short-period superlattices. J. Cryst. Growth.

[2] Jaffe M, Singh J (1987). A comparison of the electronic-properties of thin-period (InAs)(GaAs) and (InAs)(AlAs) superlattices with compositionally similar random alloys. IEEE Trans. Electron Devices.

[3] Bradshaw J, Song XJ, Shealy JR, Zhu JG, Ostergaard H (1992). Characterization by Raman-scattering, X-ray-diffraction, and transmission electron-microscopy of (AlAs)_m_ (InAs)_m_ short-period superlattices grown by migration enhanced epitaxy. J. Appl. Phys..

[4] Yao JS (2021). Large tunable bandgaps in the InAs/AlAs strain-compensated short-period superlattices grown by molecular beam epitaxy. Appl. Phys. Lett..

[5] Onda K (1998). InAlAs/InGaAs channel composition modulated transistors with InAs channel and AlAs/InAs superlattice barrier layer. IEEE Electron Device Lett..

[6] Wang WY (2021). Characteristics of thin InAlAs digital alloy avalanche photodiodes. Opt. Lett..

[7] Vazquez M (1990). Atomic layer molecular-beam epitaxy of InAs/A1As heterostructures. J. Cryst. Growth.

[8] Milekhin AG (2004). Interface phonons in InAs and AlAs quantum dot structures. Phys. Rev. B.

[9] Carles R, Saintcricq N, Renucci JB, Renucci MA, Zwick A (1980). 2nd-order Raman-scattering in InAs. Phys. Rev. B.

[10] Azuhata T, Sota T, Suzuki K (1995). Second-order Raman-spectra and lattice-dynamics in AlAs. J. Phys. Condens. Matter.

[11] Spencer GS (1994). 2nd-order Raman-spectroscopy of AlAs—A test of lattice-dynamical models. Phys. Rev. B.

[12] Liu HN (2015). Lattice vibration modes in type-II superlattice InAs/GaSb with no-common-atom interface and overlapping vibration spectra. Phys. Rev. B.

[13] Woo DH (1992). Optical characterization of coherently strained short-period superlattice (InAs) n (AlAs) n grown by molecular-beam epitaxy. Surf. Sci..

[14] Zhang SL, Klein MV, Klem J, Morkoc H (1988). Raman-scattering from confined lo phonons and dispersion-relation in GaAs/AlAs superlattices. Phys. Lett. A.

[15] Colvard C (1985). Folded acoustic and quantized optic phonons in (GaAl)as superlattices. Phys. Rev. B.

[16] Colvard C, Merlin R, Klein MV, Gossard AC (1980). Observation of folded acoustic phonons in a semiconductor superlattice. Phys. Rev. Lett..

[17] Osswald S, Mochalin VN, Havel M, Yushin G, Gogotsi Y (2009). Phonon confinement effects in the Raman spectrum of nanodiamond. Phys. Rev. B.

[18] Sood AK, Menendez J, Cardona M, Ploog K (1985). Interface vibrational-modes in GaAs-AlAs superlattices. Phys. Rev. Lett..

[19] Armelles G, Recio M, Rodriguez JM, Briones F (1989). Raman-scattering of InAs alas strained-layer superlattices. Phys. Rev. B.

[20] Milekhin A (2012). Raman scattering of InAs/AlAs quantum dot superlattices grown on (001) and (311)B GaAs surfaces. Nanoscale Res. Lett..

[21] Comas F, Trallero-Giner C, Studart N, Marques GE (2002). Interface optical phonons in spheroidal dots: Raman selection rules. Phys. Rev. B.

[22] Dimitrievska M, Fairbrother A, Perez-Rodriguez A, Saucedo E, Izquierdo-Roca V (2014). Raman scattering crystalline assessment of polycrystalline Cu2ZnSnS4 thin films for sustainable photovoltaic technologies: phonon confinement model. Acta Mater..

[23] Wang S, Dou Y, Liu H, Lin Z, Zhang H (2018). Electron momentum and energy relaxation times in wurtzite GaN, InN and AlN: A monte carlo study. J. Electron. Mater..

[24] Pavesi L, Mariotto G, Carlin JF, Rudra A, Colombo L (1994). Confinement effects on the phonon-spectrum of thin InAs InP strained single quantum-wells. Semicond. Sci. Technol..

[25] Pavesi L, Mariotto G, Carlin JF, Rudra A, Houdre R (1992). Raman-study of a single InP/InAs/InP strained quantum-well. Solid State Commun..

[26] Tran CA, Jouanne M, Brebner JL, Masut RA (1993). Effect of strain on confined optic phonons of highly strained InAs/InP superlattices. J. Appl. Phys..

[27] Tenne DA (2000). Raman study of self-assembled GaAs and AlAs islands embedded in InAs. Phys. Rev. B.

[28] Levinshtein, M. A. R., S%A Shur, M. *Handbook Series on Semiconductor Parameters*.

[29] Adachi S (1985). GaAs, AlAs, and Al_x_Ga_1-x_As—Material parameters for use in research and device applications. J. Appl. Phys..

[30] Cerdeira F, Buchenauer CJ, Cardona M, Pollak FH (1972). stress-induced shifts of first-order Raman frequencies of diamond and zinc-blende-type semiconductors. Phys. Rev. B Solid State.

[31] Yang MJ (1993). Dependence of InAs phonon energy on misfit-induced strain. Appl. Phys. Lett..

[32] Yeo W, Dimitrov R, Schaff WJ, Eastman LF (2000). The effect of As-4 pressure on material qualities of AlGaAs/GaAs heterostructures grown on (111)B GaAs substrates. Appl. Phys. Lett..

[33] Wickboldt P, Anastassakis E, Sauer R, Cardona M (1987). Raman phonon piezospectroscopy in GaAs—Infrared measurements. Phys. Rev. B.

[34] Lebedev, A. I. First-principles calculations of vibrational spectra of CdSe/CdS superlattices. (2021).

[35] Richter H, Wang ZP, Ley L (1981). The one phonon Raman-spectrum in microcrystalline silicon. Solid State Commun..

[36] Sun B (2019). Dislocation-induced thermal transport anisotropy in single-crystal group-III nitride films. Nat. Mater..

[37] Yu J (2022). Carrier localization effect in the photoluminescence of In composition engineered InAlAs random alloy. J. Lumin..

[38] Nakayama M (1985). Raman-study of GaAs-In_x_Al_1-x_As strained-layer superlattices. J. Appl. Phys..

